# Measurement of Step Angle for Quantifying the Gait Impairment of Parkinson’s Disease by Wearable Sensors: Controlled Study

**DOI:** 10.2196/16650

**Published:** 2020-03-20

**Authors:** Jingying Wang, Dawei Gong, Huichun Luo, Wenbin Zhang, Lei Zhang, Han Zhang, Junhong Zhou, Shouyan Wang

**Affiliations:** 1 Institute of Science and Technology for Brain-Inspired Intelligence Fudan University Shanghai China; 2 Key Laboratory of Computational Neuroscience and Brain-Inspired Intelligence (Fudan University) Ministry of Education Shanghai China; 3 Department of Neurosurgery The Second Hospital of Nanjing Nanjing China; 4 Department of Neurosurgery Nanjing Brain Hospital Nanjing China; 5 Department of Computer Science Virginia Tech Falls Church, VA United States; 6 Hinda and Arthur Marcus Institute for Aging Research, Hebrew SeniorLife Harvard Medical School Roslindale, MA United States

**Keywords:** Parkinson’s disease, gait, angular velocity, inertial sensor, step angle, deep brain stimulation, acute levodopa challenge test

## Abstract

**Background:**

Gait impairments including shuffling gait and hesitation are common in people with Parkinson’s disease (PD), and have been linked to increased fall risk and freezing of gait. Nowadays the gait metrics mostly focus on the spatiotemporal characteristics of gait, but less is known of the angular characteristics of the gait, which may provide helpful information pertaining to the functional status and effects of the treatment in PD.

**Objective:**

This study aimed to quantify the angles of steps during walking, and explore if this novel step angle metric is associated with the severity of PD and the effects of the treatment including the acute levodopa challenge test (ALCT) and deep brain stimulation (DBS).

**Methods:**

A total of 18 participants with PD completed the walking test before and after the ALCT, and 25 participants with PD completed the test with the DBS on and off. The walking test was implemented under two conditions: walking normally at a preferred speed (single task) and walking while performing a cognitive serial subtraction task (dual task). A total of 17 age-matched participants without PD also completed this walking test. The angular velocity was measured using wearable sensors on each ankle, and three gait angular metrics were obtained, that is mean step angle, initial step angle, and last step angle. The conventional gait metrics (ie, step time and step number) were also calculated.

**Results:**

The results showed that compared to the control, the following three step angle metrics were significantly smaller in those with PD: mean step angle (*F*_1,48_=69.75, *P*<.001, partial eta-square=0.59), initial step angle (*F*_1,48_=15.56, *P*<.001, partial eta-square=0.25), and last step angle (*F*_1,48_=61.99, *P*<.001, partial eta-square=0.56). Within the PD cohort, both the ALCT and DBS induced greater mean step angles (ACLT: *F*_1,38_=5.77, *P*=.02, partial eta-square=0.13; DBS: *F*_1,52_=8.53, *P*=.005, partial eta-square=0.14) and last step angles (ACLT: *F*_1,38_=10, *P*=.003, partial eta-square=0.21; DBS: *F*_1,52_=4.96, *P*=.003, partial eta-square=0.09), but no significant changes were observed in step time and number after the treatments. Additionally, these step angles were correlated with the Unified Parkinson's Disease Rating Scale, Part III score: mean step angle (single task: *r*=–0.60, *P*<.001; dual task: *r*=–0.52, *P*<.001), initial step angle (single task: *r*=–0.35, *P*=.006; dual task: *r*=–0.35, *P*=.01), and last step angle (single task: *r*=–0.43, *P*=.001; dual task: *r*=–0.41, *P*=.002).

**Conclusions:**

This pilot study demonstrated that the gait angular characteristics, as quantified by the step angles, were sensitive to the disease severity of PD and, more importantly, can capture the effects of treatments on the gait, while the traditional metrics cannot. This indicates that these metrics may serve as novel markers to help the assessment of gait in those with PD as well as the rehabilitation of this vulnerable cohort.

## Introduction

Gait impairment, which is induced by diminished locomotor control [1], is highly prevalent in Parkinson’s disease (PD) [2]. People with PD often suffer from multiple symptoms of gait impairment, including freezing of gait, hesitation at the beginning of walking, festination, and difficulty in stopping at the end of walking [3]. These gait impairments often lead to increased risk of falls, loss of functional independence in daily life, and even increased risk of morbidity and mortality [4]. Studies have linked the subtle changes in PD gait to other diseases and conditions such as dementia and history of head trauma [5]. It is thus of great clinical significance to measure and characterize the gait in PD, which will ultimately provide insights into the pathophysiology of PD and help optimize the therapeutic strategies such as deep brain stimulation (DBS).

Multiple instruments including motion capture systems [6], pressure mats [7], wearable sensors [8,9], and smartphone apps that use an accelerometer and gyroscope [10] have been developed to quantify the gait metrics of patients with PD [11]. Wearable sensors have become a rapidly growing solution to quantitatively assess the symptoms of PD, allowing more convenient testing protocol and remote and longer-term assessment and tracking of the functionality of people suffering from PD [12]. The wearable sensor can provide multiple spatiotemporal gait metrics of great clinical importance in PD assessment [12]. For example, the step time (or stride interval) [13,14] and step number (or step count) [13,15] can quantify the severity of locomotor dysfunction in those with PD and distinguish between the stages of PD. Additionally, gait speed [16-18], stride length [16,18], and cadence [17,18] can help identify abnormalities caused by PD and evaluate the efficacy of treatment.

Many impairments in PD, such as festinating gait, shuffling gait, hesitation, and start-stop difficulty have a visible resemblance (ie, the angles in the sagittal plane, the anatomical boundary dividing the left and right parts of the body, of the leg will shrink while walking). Several studies [19,20] observed significant correlation between the changes in the sagittal plane characteristics of the lower limbs and the clinical score, suggesting that such angular changes in the sagittal plane can provide meaningful clinical evaluations of PD. However, less is known about the angular characteristics of the sagittal plane gait in people with PD.

In this study, we aimed to explore if the angular characteristics of gait, especially in the sagittal plane, are sensitive to the clinical and functional characteristics of PD by measuring the step angles using wearable sensors fixed on the ankles. The mean step angle, step angle within the initiation of walking, and step angle within the end period of walking were measured. We hypothesize that the step angle would be significantly different between people with PD and those without PD (ie, control group); would be sensitive to the treatment (ie, medication and DBS) and cognitive demands; and would be significantly correlated to the Unified Parkinson's Disease Rating Scale, Part III (UPDRS-III) score.

## Methods

### Participants

A total of 30 participants with PD and 17 age-matched participants without PD were recruited. All participants provided written informed consent as approved by the institutional review committee of the Nanjing Brain Hospital. The inclusion criteria for the PD cohort were: having idiopathic PD as diagnosed by experienced clinicians based on the Chinese Diagnostic Criteria of Parkinson's Disease (2016), a surgery plan of DBS within 2 months, and able to stand and walk unassisted for more than 10 minutes. The exclusion criteria were: being younger than 40 years, having any other major neurological diseases (eg, stroke, dementia), and having ongoing psychiatric disturbances such as hallucinations.

### Experimental Protocol

Before and after the treatments (ie, the acute levodopa challenge test [ALCT] or DBS), each participant in the PD group completed one 10-meter walking test for each of the following conditions: walking normally (ie, single task) and walking while performing a cognitive task (ie, dual task). The cognitive task was the serial subtraction of 3 or 7 from a random three-digit number. The UPDRS-III was also completed before and after the treatments and was used to assess the severity of PD. The healthy cohort completed one study visit consisting of the same single and dual task walking trials.

All trials were completed in the same room. One study personnel stood at the end of the walkway, and their position was fixed. No markers were used in the room, as the marker may give a cue to the participants in the PD group, and the pathway was not approaching a wall or doorway, which could interfere with participants’ gait. Each participant stood in front of a wooden chair on one side of the pathway at the beginning of the trial and was instructed to walk along the pathway and stop at the same position as the study personnel. During each walking trial, two wearable sensors were used and attached to each ankle. The kinematic signals of walking including the angular velocity were then recorded and used to quantify the gait metrics.

### Wearable Sensor

**Figure 1:**
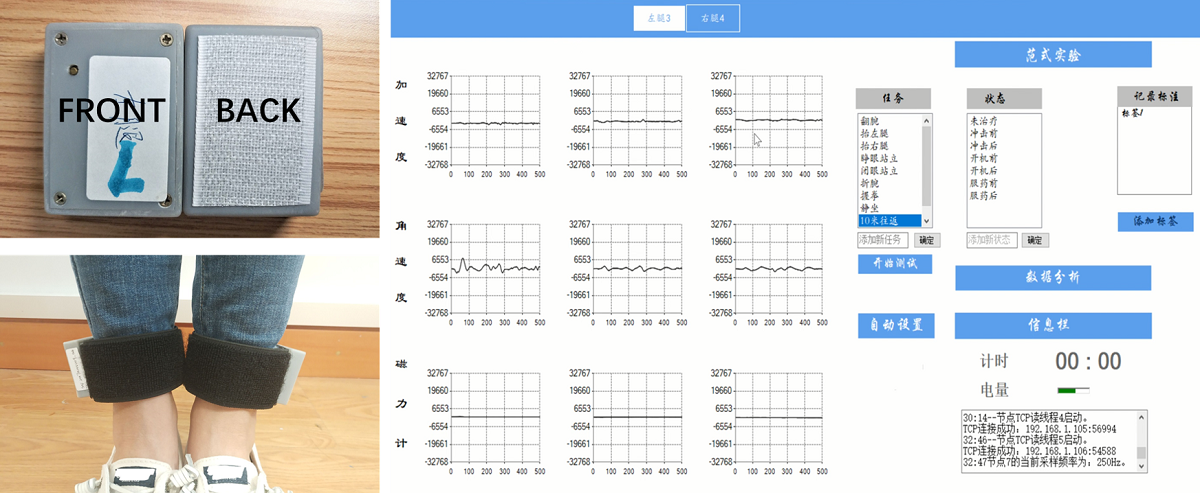
Two sensors to measure the angular velocity of each ankle separately and the software interface of the sensors.

The wearable sensors (Figure 1) used in the study were developed by our team and embedded with inertial measurement units (chip MPU-9250, IvenSense Inc, San Jose, CA). The sensors captured triaxial acceleration, angular velocity, and magnetic field intensity signals. The size of the sensors was 52 mm by 37 mm by 13 mm, and they each weighed 26.3 g. The average operating current of the sensors was 38.1 mA allowing for 13 hours of continuous recording. The sampling rate was 100 Hz, and the range of measurement for the gyroscope was ±1000 °/sec with a resolution of 0.06 °/sec/least significant bit.

### Acute Levodopa Challenge Test and Deep Brain Stimulation

The ALCT was performed in the morning using the established formulation to observe the clinical improvement in the PD group following withdrawal of all antiparkinsonian medication and overnight fasting [21]. The ALCT was used in clinics to screen patients who could use DBS based on their response to medication [21], that is if patients’ UPDRS-III score reduced by more than 30% after the ALCT, they would benefit from DBS.

DBS is a type of neurosurgical surgery that sends electrical impulses through implanted electrodes to specific brain nuclei and alleviates the burden of multiple movement disorders such as tremors in PD [22]. In this study, the DBS surgery was conducted after a minimum 1-week break from the ALCT. The pulse of the DBS was set up using the width of 60 μs and a frequency of 130 Hz. On the DBS visit, which was during participants’ perioperative period, participants completed the walking test with the DBS off and on without taking any medicine.

### Data Processing

**Figure 2:**
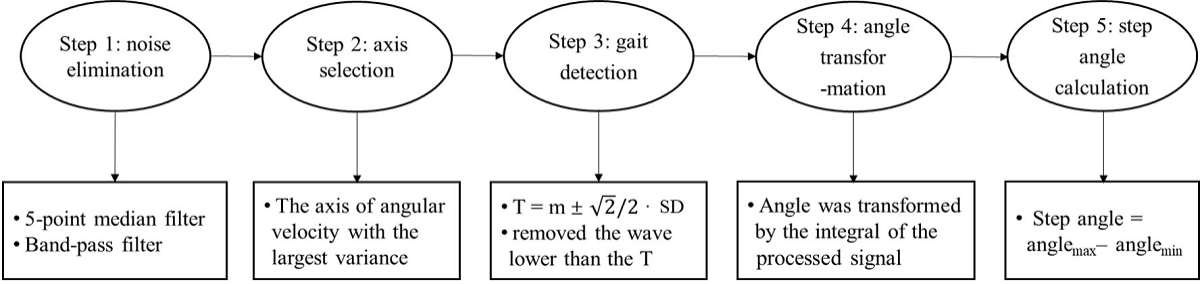
The procedure of data processing.

**Figure 3:**
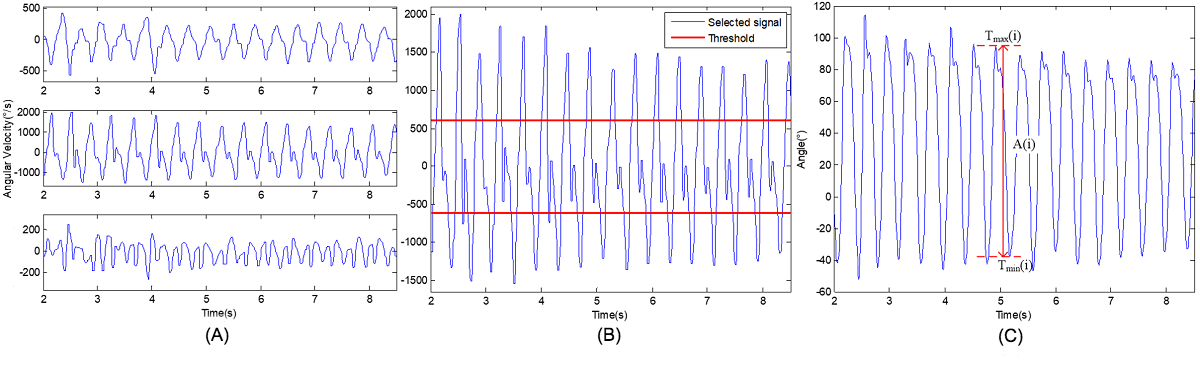
One signal in different steps of data processing: (A) 3-axis angular velocity signals; (B) the selected signal Y and thresholds; (C) the calculation of step angle.

Figure 2 shows the pipeline of the data processing. First, we removed isolated noise points by using a 5-point median filter, and a band-pass filter was applied to remove fluctuations of frequency greater than 12 Hz and lower than 0.1 Hz. Second, the angular velocity of the axis with the largest variance was selected (Figure 3A). We observed that the angular changes in the sagittal plane during walking were obvious and much greater than the changes in other planes, so we focused on the angular characteristics of gait in this plane. Third, to divide gait cycles accurately, a threshold was set to exclude the influence of small displacements of the legs, and only the peaks and valleys that were greater than the threshold (ie, the heel strikes or toe-offs) were identified and used. The threshold was calculated as the mean of this signal plus or minus √2 / 2 SD (Figure 3B). The next step is the transformation from angular velocity to the degree of angle. The degree of angle was converted by the integral of the processed velocity signal. Finally, the step angle of each step was calculated as the difference of angle, A_(i)_, between a peak of the wave, that is the angle of the maximum threshold=T_max(i)_, and the adjacent valley, that is the angle of the minimum threshold=T_min(i)_, in the angle degree series (Figure 3C).

### Gait Metrics

Five gait metrics were calculated from the angular velocity signal, including 2 conventional gait metrics (step time and step number) and 3 new metrics (mean step angle, initial step angle, and last step angle). The definition of step angle is the angular change of the ankle in the sagittal plane within 1 gait cycle. The definitions of all 5 gait metrics are provided in Textbox 1. All the metrics here were obtained by averaging the left and right legs.

**Gait metrics and their definitions.**

**Conventional metrics**

- Step time: the mean time to complete steps in 1 walking trial
- Step number: the total number of steps in 1 walking trial

**New metrics**

- Mean step angle: the arithmetic average of step angles in 1 walking trial
- Initial step angle: the angle of the first step in 1 walking trial
- Last step angle: the angle of the last step in 1 walking trail

### Data Analysis

The statistical analysis was performed using SPSS 20 (IBM Corp, Armonk, NY). To examine the effects of group and task on step angle, 2-factor multivariate analyses of variance (MANOVAs) were used. The outcomes measured before the ALCT were used as the baseline outcomes of the PD group, as there was no influence for the implanted DBS electrodes. The dependent variables were mean step angle, initial step angle, and last step angle measured before the treatment (ie, baseline) in each model, and the model effects included the groups (ie, PD vs healthy), tasks (ie, single vs dual task), and their interactions. Similar models were also used to examine the effects of group and task on the two traditional metrics (ie, step time and number). To explore the effects of the ALCT on the UPDRS-III score and gait metrics, a 2-way MANOVA was used. The dependent variables were the 5 gait metrics and the UPDRS-III score. The model effects were time (before and after), task condition (single and dual task), and their interaction. The effects of DBS were then examined using the same models. If the interaction effect was significant, the analysis of simple effect was applied to further analyze the differences of the 5 gait metrics (dependent variables) between two levels of one independent variable while fixing another factor. The Bonferroni correction was used for multiple comparisons, where the significance level was set at *P*<.01. Partial eta-square was calculated for the effect size of the MANOVA. For partial eta-squares, 0.01, 0.06, and 0.14 were considered as small, medium, and large effect sizes, respectively [23].

Then a partial correlation analysis adjusted for age, gender, and status of treatment was used to explore the association between the UPDRS-III score and gait metrics at baseline. In addition, the independent-samples *t* test (two-tailed) and the Mann-Whitney U test were used to examine the differences in age and gender, respectively, between the two groups. A Bonferroni correction was used for the multiple comparisons, and the significance was set as *P*<.01.

The significance level was set at *P*<.05, not including those corrected by the Bonferroni correction.

## Results

### Demographics and Clinical Characteristics

A total of 18 participants in the PD group completed the tests at baseline (ie, before the ALCT), after the ALCT, and after the DBS surgery. In addition, 25 participants in the PD group completed the tests when the DBS was on and off. All participants completed the study. Table 1 shows their demographic and clinical information. No significant differences in age (*F*_2,57_=0.18, *P*=.84) or gender (χ^2^_2_=1.66, *P*=.44) between the PD cohort and non-PD control group were observed. There were no significant differences of disease duration (t_36_=0.16, *P*=.88), the Hoehn-Yahr stage (t_32_=0.04, *P*=.97), and UPDRS-III score (t_22_=–0.24, *P*=.81) between the ALCT and DBS groups.

**Table 1:** Demographics and clinical characteristics of participants.

Characteristics	Non-PD^a^ (N=17)	PD with ALCT^b^ treatment (N=18)	PD with DBS^c^ treatment (N=25)
Gender, female, n (%)	8 (47%)	12 (67%)	16 (64%)
Age (years), mean (SD)	62.4 (7.1)	63.6 (5.9)	63.4 (6.9)
Disease duration (years), mean (SD)	n/a^d^	10.07 (2.65)	9.92 (3.08)
H-Y^e^ stage, mean (SD)	n/a	3.29 (0.87)	3.28 (0.83)
UPDRS-III^f^ score, mean (SD)	n/a	39.09 (13.33)	40.31 (11.65)

^a^PD: Parkinson’s disease.

^b^ALCT: Acute Levodopa Challenge Test.

^c^DBS: Deep Brain Stimulation.

^d^Not applicable.

^e^H-Y: Hoehn-Yahr.

^f^UPDRS-III: Unified Parkinson's Disease Rating Scale, Part III.

### Comparison of Gait Metrics Between People With Parkinson’s Disease and Controls

A significant main effect of group was observed, but no significant difference was observed for the effect of task and their interaction (Table 2). The mean step angle (*F*_1,48_=69.75, *P*<.001, partial eta-square=0.59), initial step angle (*F*_1,48_=15.56, *P*<.001, partial eta-square=0.25), and last step angle (*F*_1,48_=61.99, *P*<.001, partial eta-square=0.56) in participants with PD were all significantly smaller than healthy people. Similar results were shown in those conventional metrics that the PD cohort had significantly larger step times (*F*_1,48_=7.52, *P*=.009, partial eta-square=0.14) and more step numbers (*F*_1,48_=12.05, *P*=.001, partial eta-square=0.20) than the control.

**Table 2:** Gait metrics measured at baseline in participants with Parkinson’s disease and healthy participants.

Metrics	non-Parkinson’s disease, mean (SD)		Parkinson’s disease, mean (SD)	
	Single	Dual	Single	Dual
Step time (sec/step)	0.89 (0.05)	0.97 (0.09)	1.04 (0.37)	1.2 (0.41)
Step number	30.62 (9.05)	34.34 (9.86)	50.95 (59.48)	89.94 (65.44)
Mean step angle (^o^)	63.33 (7.32)	61.85 (9.01)	30.71 (20.36)	26.87 (21.06)
Initial step angle (^o^)	34.50 (4.80)	32.88 (6.35)	33.71 (5.58)	20.79 (16.99)
Last step angle (^o^)	64.33 (15.20)	60.82 (14.21)	28.54 (15.63)	27.12 (16.56)

### Effects of Acute Levodopa Challenge Test and Deep Brain Simulation on the Gait Metrics

We observed that the UPDRS-III score significantly decreased after the treatments (ie, the ALCT and DBS) compared to the score before the treatments (ACLT:t_11_=–7.81, *P*<.001; DBS:t_15_=–15.22, *P*<.001).

#### Acute Levodopa Challenge Test

A significant main effect of time (before the ALCT vs after the ALCT) was observed, but no significant main effect of task and their interaction were observed (Table 3). Specifically, the mean step angle (*F*_1,38_=5.77, *P*=.02, partial eta-square=0.13) and last step angle (*F*_1,38_=10, *P*=.003, partial eta-square=0.21) after the ALCT were greater than that at baseline, while the initial step angle (*F*_1,38_=2.55, *P*=.12, partial eta-square=0.06) was not significantly changed. No significant changes were observed in the conventional metrics step number (*F*_1,38_=4.33, *P*=.05, partial eta-square=0.10) and step time (*F*_1,38_=2.01, *P*=.17, partial eta-square=0.05) after the ALCT.

**Table 3:** Gait metrics before and after acute levodopa challenge test.

Metrics	Before ALCT^a^, mean (SD)		After ALCT, mean (SD)	
	Single task	Dual task	Single task	Dual task
Step time (sec/step)	1.04 (0.37)	1.20 (0.41)	0.96 (0.21)	1.01 (0.24)
Step number	50.95 (59.48)	89.94 (65.44)	32.21 (28.8)	46.77 (34.26)
Mean step angle (^o^)	30.71 (20.36)	26.87 (21.06)	46.48 (18.87)	40.63 (18.82)
Initial step angle (^o^)	20.79 (16.99)	21.26 (16.93)	32.99 (17.18)	26.03 (16.92)
Last step angle (^o^)	28.54 (15.63)	27.12 (16.56)	43.91 (17.37)	43.06 (13.66)

^a^ALCT: acute levodopa challenge test.

#### Deep Brain Stimulation

A significant main effect of time (DBS off vs DBS on) was observed, but not in the main effect of task and their interaction (Table 4). Specifically, mean step angle (*F*_1,52_=8.53, *P*=.005, partial eta-square=0.14) and last step angle (*F*_1,52_=4.96, *P*=.003, partial eta-square=0.09) with DBS on were both significantly greater than those with DBS off, but no significant changes in initial step angle (*F*_1,52_=2.94, *P*=.09, partial eta-square=0.05) were observed. In conventional metrics, step time (*F*_1,52_=5.59, *P*=.02, partial eta-square=0.1) had a marginally significant decrease when DBS was on, and no significant improvement was observed in step number (*F*_1,52_=1.33, *P*=.25, partial eta-square=0.03).

**Table 4:** Gait metrics under deep brain stimulation off and deep brain stimulation on conditions.

Metrics	Deep brain stimulation off, mean (SD)		Deep brain stimulation on, mean (SD)	
	Single task	Dual task	Single task	Dual task
Step time (sec/step)	1.24 (0.65)	1.21 (0.48)	0.91 (0.23)	0.98 (0.28)
Step number	50.64 (82.6)	66.69 (41.31)	36.37 (23.01)	50.89 (23.35)
Mean step angle (^o^)	34.36 (17.77)	28.72 (17.52)	45.88 (16.61)	43.83 (16.25)
Initial step angle (^o^)	21.73 (12.59)	18.32 (9.55)	26.34 (12.93)	25.02 (13.61)
Last step angle (^o^)	33.59 (21.71)	25.47 (17.43)	40.55 (21.44)	42.53 (19.45)

### Relationships Between Gait Metrics and Unified Parkinson's Disease Rating Scale, Part III

Table 5 presented the association between the gait metrics and the UPDRS-III score. Age, gender, and condition were controlled in this analysis, and the degree of freedom in single and dual walking tasks were 58 and 51, respectively. The mean, initial, and last step angles in both single and dual walking tasks were all significantly correlated with the UPDRS-III score (*r*>–0.35, *P*<.01). A weaker correlation was observed between the step time and number and the UPDRS-III scores. The scatter plots of the UPDRS-III score and gait metrics are shown in Multimedia Appendix 1.

**Table 5:** Partial correlation analysis between the Unified Parkinson's Disease Rating Scale, Part III score and gait metrics.

Metrics	Single task, *r*	*P* value	Dual task, *r*	*P* value
Step time (sec/step)	0.32	.01	0.42	.002
Step number	0.18	.17	0.35	.01
Mean step angle (^o^)	–0.60	<.001	–0.52	<.001
Initial step angle (^o^)	–0.35	.006	–0.35	.01
Last step angle (^o^)	–0.43	.001	–0.41	.002

## Discussion

This study demonstrated that the angular characteristics of gait, as quantified using the step angles measured in the sagittal plane of the lower limbs, are sensitive to PD and the treatments (ie, the ALCT and DBS) and consistent in different cognitive conditions. Specifically, we observed that the mean, initial, and last step angle were significantly smaller in the PD cohort compared to the healthy cohort and similar between single and dual task conditions. In addition, the mean step angle and last step angle were significantly increased after treatments and were associated with the UPDRS-III score. These results suggest that these novel angular metrics are sensitive to the severity of PD and captures the effects of treatments on gait in people with PD, as greater step angles reflected better locomotor control of walking.

We observed that the new angular metrics were smaller in those with PD across the conditions (single and dual task) compared to the healthy cohort and significantly correlated with the UPDRS-III total score. Participants with greater UPDRS-III scores had smaller step angles. The commonly used gait metrics focused more on the temporal (eg, stride time) or spatial (eg, stride length) characteristics of gait, but the musculoskeletal rotation of the extremities is also important for the completion of 1 gait cycle. The step angle captures the angular change (ie, rotation) in the sagittal plane of lower limbs during walking. The diminished locomotor control in PD may induce more variance in the rotation and thus impair gait patterns (eg, incomplete gait cycles). These step angles measuring the subtle changes in the musculoskeletal rotation may thus help quantify the gait impairments in PD. The initial and last step angles, for example, can particularly help assess the start hesitation and stop difficulty in PD. It should also be noted that compared to those traditional metrics (step time, step number), which have been proved effective in reflecting gait impairments in patients with PD [13], the effect size of the angular metrics is much larger, indicating that these new metrics are more sensitive to the effects of PD on gait. Future longitudinal studies are needed to explore how these angular characteristics of gait change along with the progress of PD.

These step angles were sensitive to both the ALCT and DBS within the PD cohort. This is consistent with the results of previous studies using other gait metrics [24]. Specifically, the mean and last step angles were increased when DBS was working, and the mean and last step angles were improved after the ALCT. However, no significant improvement was observed in those traditional metrics. These results support that the subthalamic nucleus (STN)-DBS treatment improves gait performance in PD [25-27]; however, some studies [28,29] reported the effects of STN-DBS on gait are less successful and may even lead to an aggravation of freezing of gait and imbalance. The results here suggested that step angle metrics may capture the subtle changes of gait and the acute effects of the treatment on gait. The effect size of the ALCT was larger than that of DBS, which is in support of previous studies showing a lower benefit on gait velocity and stride length by DBS [30] and a higher benefit on cadence by levodopa [31]. However, this still suggests that the STN-DBS has less effectiveness on gait compared to the levodopa treatment.

Previous studies have shown that the performance of simultaneous cognitive tasks compromised gait in people with and without PD [32,33] with a decrease in stride time and an increase in stride frequency [34]. However, this study showed that compared to single-task walking, no statistically significant change in step time, step number, and step angles were observed in dual task walking. One potential reason may be the relatively small sample size in this piloting study. However, a greater change in step angle from single to dual task walking in those with PD (average change of 5°) compared to the control cohort (smaller difference of only 2°) was observed, indicating that PD diminished the capacity of walking control in the dual task condition.

The results of this study further provided evidence that wearable inertial sensors can help advance the traditional measurements of gait and other neurophysiological and biomechanical signals (eg, the center of pressure of a human body) into a quicker, convenient protocol [35]. Traditional laboratory or clinical tests assessing the gait are dependent upon expensive and nonportable equipment and well-trained study personnel, which presents a challenge for people living in rural areas or areas distant from hospitals. This type of wearable sensor provides a novel approach to these populations for assessing their gait and other health information. It should be noted that the intersubject variance of gait metrics was much greater (ie, larger SD) in the PD cohort compared to the healthy cohort in this study, indicating that subtle characteristics of gait vary across the PD population. Previous studies also showed that within one person, the day-to-day variance in gait and physical activity was high, and such variance was associated with their health status, such as cognitive impairments [36]. Taken together, the measurement of gait and other health information using wearable sensors facilitates the high-frequency monitoring in those vulnerable populations, which will ultimately help in clinical diagnosis and disease prevention.

The small sample size and lack of cognition examination are two limitations of this study. The difference in cognitive function, which may influence the gait, between the two groups was not included in the analyses. Future studies with a larger sample size and assessment of cognitive function, using the Montreal Cognitive Assessment or the Mini Mental State Examination, are needed to examine and confirm the findings in this pilot study. The flexibility of the knee also contributes to the control of musculoskeletal rotation of gait, and thus the angular change of the knee may also provide important information about locomotor control. Future work using an electronic goniometer is needed to measure the angular characteristics of the knee during walking. Nevertheless, this study proposed novel metrics to quantify the angular characteristics of gait and demonstrated that the step angle metrics are sensitive to the effects of PD on gait, disease severity, and the effects of treatments on gait, which may serve as novel markers that help the management of PD.

## Appendix

Multimedia Appendix 1 Scatter plots of the UPDRS-III score and gait metrics.

## Abbreviations

ALCT: acute levodopa challenge test
DBS: deep brain stimulation
MANOVA: multivariate analysis of variance
PD: Parkinson’s disease
STN: subthalamic nucleus
UPDRS-III: Unified Parkinson's Disease Rating Scale, Part III.
