# Supplementary figures and images for: Measurement of Step Angle for Quantifying the Gait Impairment of Parkinson’s Disease by Wearable Sensors: Controlled Study
Source: JMIR Mhealth Uhealth. 2020 Mar 20;8(3):e16650. doi: 10.2196/16650 (PMC7125438; doi:10.2196/16650)

**Single Task**

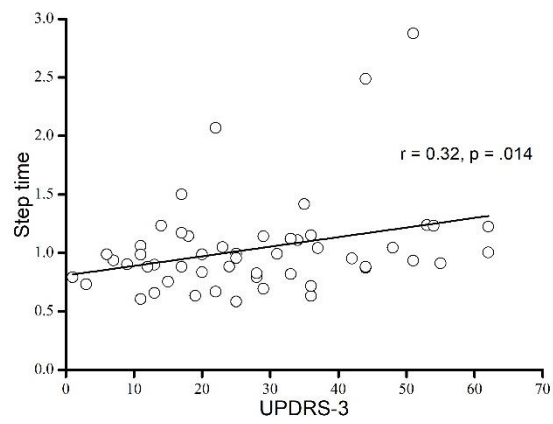

**Dual Task**

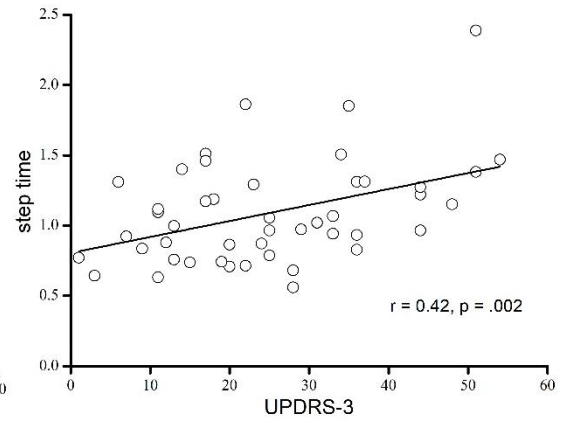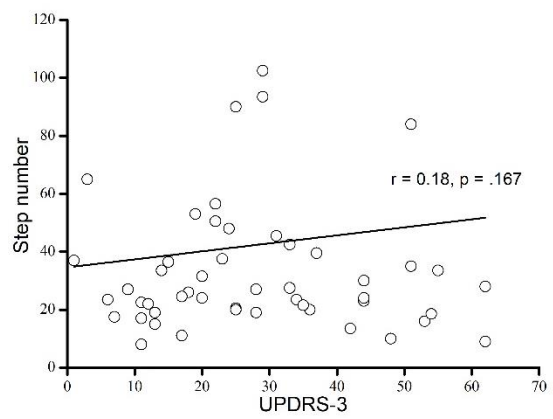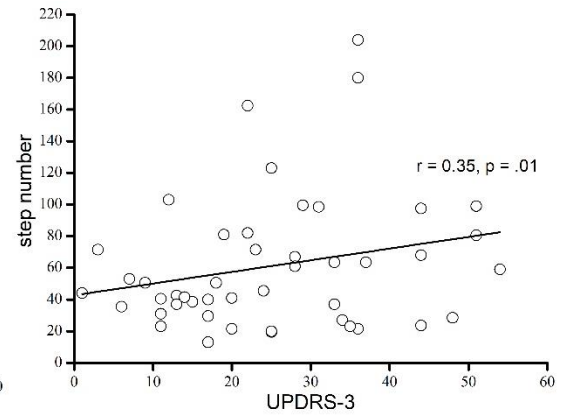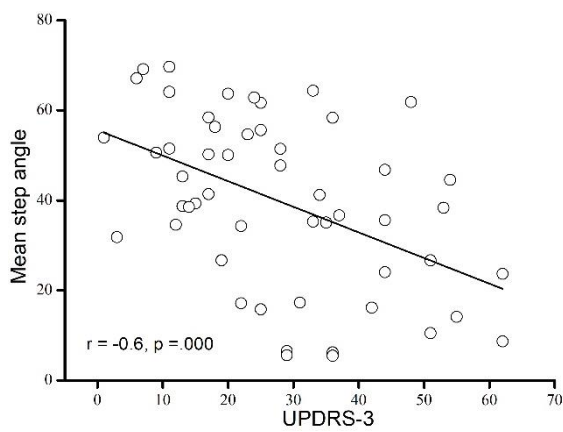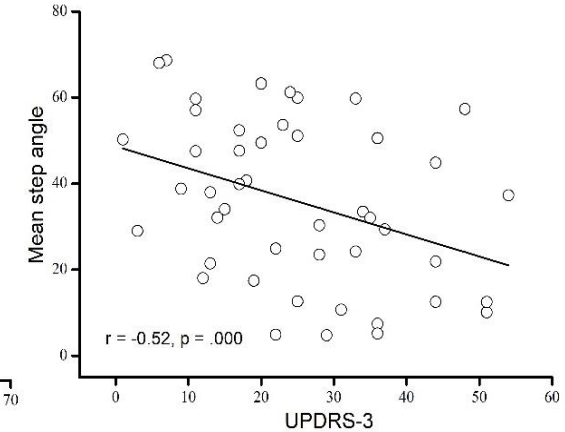

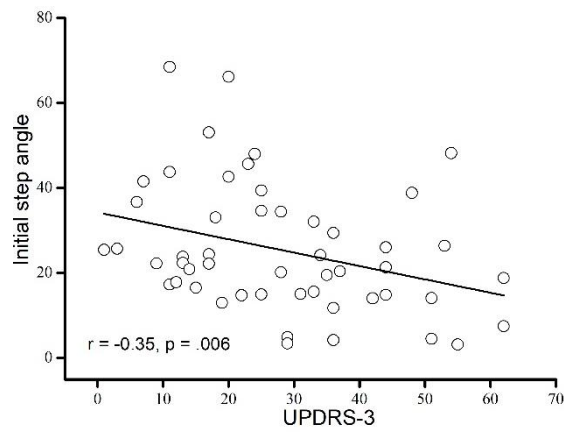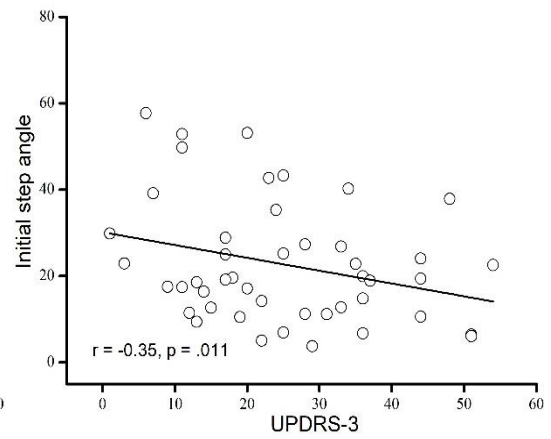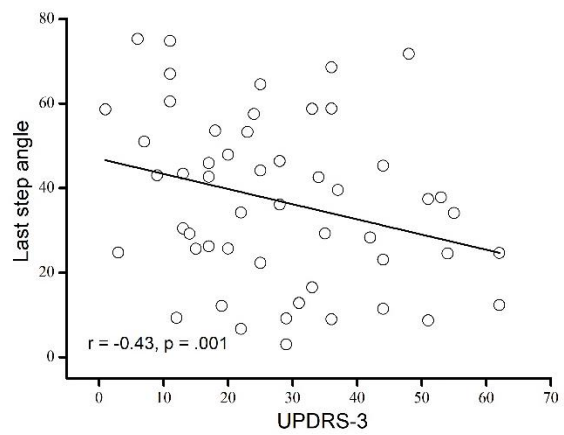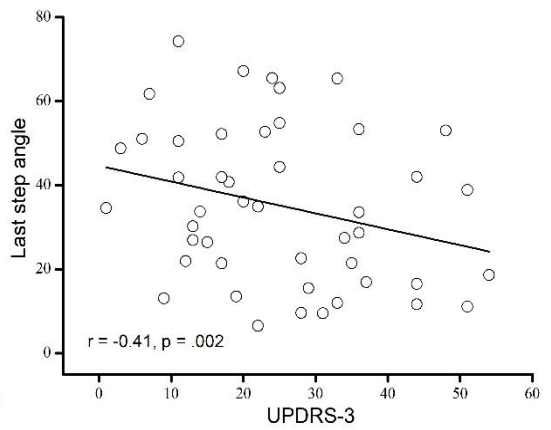

Supplement: Multimedia Appendix 1 [file mhealth_v8i3e16650_app1.pdf]
